# Evaluating the Effectiveness of Probiotic and Multivalent Vaccination Strategies in Mitigating Bacterial Chondronecrosis with Osteomyelitis Lameness Using a Hybrid Challenge Model

**DOI:** 10.3390/ani15040570

**Published:** 2025-02-16

**Authors:** Amanda Anthney, Khawla Alharbi, Ruvindu Perera, Anh Dang Trieu Do, Andi Asnayanti, Reginald Onyema, Sara Reichelt, Antoine Meuter, Palmy R. R. Jesudhasan, Adnan A. K. Alrubaye

**Affiliations:** 1 Center of Excellence for Poultry Science, University of Arkansas, Fayetteville, AR 72701, USA; apanthne@uark.edu (A.A.); ka030@uark.edu (K.A.); rperera@uark.edu (R.P.); ad086@uark.edu (A.D.T.D.); aasnayan@uark.edu (A.A.); palmy.jesudhasan@usda.gov (P.R.R.J.); 2 Cell and Molecular Biology Program, University of Arkansas, Fayetteville, AR 72701, USA; 3 Aviagen North America (NA), Huntsville, AL 35806, USA; ronyema@aviagen.com (R.O.); sreichelt@aviagen.com (S.R.); 4 Animal and Plant Health & Nutrition, Novonesis, 2970 Hørsholm, Denmark; antme@novonesis.com; 5 Poultry Production and Product Safety Research Unit, United States Department of Agriculture-Agricultural Research Service (USDA-ARS), Fayetteville, AR 72701, USA

**Keywords:** lameness, broilers, BCO, probiotic, eBeam-inactivated vaccine

## Abstract

Bacterial chondronecrosis with osteomyelitis (BCO) is caused by several bacteria, including *Salmonella*, *Staphylococcus* spp., *Escherichia coli*, *Enterococcus* spp., and *Mycoplasma* spp., and BCO is a significant animal health and welfare issue in broiler production, causing 1–2% of bird condemnation at marketing age and resulting in annual losses of tens of millions of dollars. This study evaluated the efficacy of a probiotic program alone and combined with a multivalent electron beam (eBeam)-inactivated vaccine in reducing BCO lameness. The probiotic program included an *Enterococcus faecium* spray (*E. faecium* 669, at 2 × 10^9^ CFU/bird) at hatch and a triple-strain *Bacillus*-based product (*B. subtilis* 597, *B. subtilis* 600, and *B. amyloliquefaciens* 516 at 1 × 10^9^ CFU/bird/day) in drinking water from day 1 to day 56. An aerosol transmission challenge model simulated commercial bacterial exposure. Birds were divided into five groups: a positive control (PC) group (T1) and a negative control (NC) group (T2) receiving no treatment and three treatment groups receiving the probiotic program (T3), the multivalent vaccine (T4), or both the probiotic program and the multivalent vaccine (T5). Data analyzed via ANOVA (*p* < 0.05) showed T3, T4, and T5 had significantly lower lameness (43.7%, 40.3%, and 40.7%) than T2 (71.0%) and T1 (83.0%). T5 resulted in reductions comparable to T4, indicating no significant synergistic effect. These findings show that probiotics alone or with a vaccine effectively mitigate BCO lameness, enhance broiler welfare, and reduce economic losses.

## Simple Summary

Bacterial chondronecrosis with osteomyelitis (BCO) is caused by *Salmonella*, *Staphylococcus* spp., *Escherichia coli*, *Enterococcus* spp., and *Mycoplasma* spp. It is a leading cause of broiler lameness, resulting in economic losses and welfare concerns. BCO occurs when bacteria enter the bloodstream and colonize bone tissue. It is exacerbated by rapid growth, mechanical stress (e.g., repetitive pressure on hock joints and footpads), uneven flooring, or excessive body weight in commercial settings. This study tested the effectiveness of a probiotic program and an inactivated vaccine in reducing BCO lameness. The probiotic program included an *Enterococcus faecium* spray at hatch and a triple-strain *Bacillus*-based product in drinking water, while the vaccine was an electron beam-inactivated multivalent. Using a wire-floor aerosol transmission challenge model, results showed probiotics, vaccines, and their combination reduced lameness by ~30% compared to untreated controls, improving welfare and reducing losses.

## 1. Introduction

The global poultry industry has undoubtedly become a cornerstone of animal protein production, effectively addressing consumer demand for affordable and efficient meat sources [1]. Importantly, as the leading livestock production sector, broiler chickens play a crucial role in meeting this demand because of the significant advancements in genetic selection, nutrition, and management practices. Consequently, these advancements have led to remarkable growth rates and improved feed conversion efficiency in modern broilers. Thus, global poultry production continues to rise consistently [1,2]. However, this rapid progress has simultaneously introduced several challenges, particularly concerning animal welfare and health. Among the most pressing issues is lameness, a multifactorial condition that significantly affects broiler productivity, welfare, and economic viability. Specifically, bacterial chondronecrosis with osteomyelitis (BCO) lameness has emerged as a predominant cause of mobility impairment in broilers, with its prevalence increasing globally [3,4].

The rapid growth of broilers creates significant structural challenges for their skeletal system. This is driven by targeted genetic selection for increased body weight and muscle mass. Consequently, this accelerated development places substantial stress on the bones, which can lead to various complications [4,5,6]. As broilers grow, their bones often fail to develop at a comparable rate to their muscle mass. Thus, it creates mechanical stress on the skeletal framework. This disparity can lead to microfractures and clefts in the bones, particularly in the femur and tibia, which are critical for mobility [4,5,6]. These microfractures further create an environment that promotes bacterial colonization, and opportunistic pathogens can infiltrate these weakened areas, leading to conditions like bacterial chondronecrosis with osteomyelitis (BCO) lameness [4,6]. Notably, BCO lameness is characterized by necrotic lesions in the proximal growth plates of the femora and tibiae, typically resulting from bacterial infections that colonize microfractures in these rapidly growing bones. Furthermore, the accelerated growth rates of modern broiler strains exacerbate this condition, as the development of their skeletal system often lags behind their dramatic musculoskeletal gains [7,8]. Common pathogens implicated in BCO include *Staphylococcus* spp., *Enterococcus* spp., and *Escherichia coli*, which translocate hematogenously from compromised respiratory or gastrointestinal tracts to infect growth plates [9,10]. BCO lameness poses significant challenges, impacting both animal welfare and productivity. For instance, this condition often leads to higher culling rates and results in decreased growth performance, reduced feed efficiency, and overall lower productivity in affected flocks [11]. Making matters worse, the industry still faces notable gaps in early diagnostic capabilities and effective therapeutic interventions for BCO despite its dire economic and welfare ramifications, making prevention a critical focus area.

In recent years, increasing concerns about antibiotic resistance and negative consumer perceptions of antibiotic use have consequently led to restrictions on antibiotics in poultry production. As a result, this has shifted the focus to sustainable alternatives such as probiotics, which offer a natural and effective means to support broiler health and performance [12,13]. Probiotics are defined as live microorganisms, primarily bacteria or yeast, that provide health benefits to the host when administered in adequate amounts. These beneficial microorganisms’ function by promoting a balanced gut microbiome, enhancing intestinal integrity, and modulating immune responses [12,13]. For instance, *Enterococcus faecium*, a Gram-positive bacterium commonly used as a probiotic, has demonstrated significant potential in promoting gut health and reducing lameness in broilers. Specifically, when applied as a spray at hatch, probiotics based on a specific *E. faecium* strain can rapidly colonize the gut, fostering the growth of beneficial microbiota, reducing inflammation, and strengthening the intestinal epithelial lining [14]. Consequently, this enhanced gut barrier prevents harmful pathogens from translocating into the bloodstream, thereby reducing the likelihood of systemic infections like BCO [15,16,17,18]. Moreover, *E. faecium* promotes villus development in the intestines, improving nutrient absorption critical for bone development and optimal growth [19,20,21]. It also enhances immune function by activating T-helper cells and cytotoxic T lymphocytes, which play crucial roles in combating infections and fostering robust adaptive immunity [15,22]. Similarly, *Bacillus*-based probiotics, such as products including specific *Bacillus subtilis* and *Bacillus amyloliquefaciens* strains, have also emerged as valuable tools in improving broiler performance and health. Notably, these probiotics reshape the gut microbiota by promoting beneficial microbial pathways and suppressing harmful ones linked to protein fermentation [23,24]. Furthermore, they reduce the incidence of necrotic enteritis caused by *Clostridium perfringens*, a common intestinal disease in poultry [25].

Additionally, *Bacillus* strains contribute to immune modulation by increasing levels of cytokines and immune cells, which are essential for controlling infections and maintaining overall health [26]. These strains also provide metabolic advantages, including enhanced nutrient utilization and improved feed conversion ratios, contributing to better growth performance [21]. Consequently, by maintaining a stable and diverse gut microbiome, *Bacillus* probiotics prevent dysbiosis and associated health issues, ensuring healthier and more productive broiler flocks [21]. Together, *E. faecium* and *Bacillus*-based probiotics not only improve intestinal health and immune function but also align with sustainable and welfare-focused poultry production practices. Thus, these benefits make probiotics a promising alternative to antibiotics, offering a practical solution to enhancing broiler health, minimizing disease, and improving productivity.

Based on this work, our previous research successfully introduced an electron-beam (eBeam)-killed *Staphylococcus* vaccine (the multivalent eBeam vaccine), which significantly reduced the incidence of lameness in broilers [27]. The eBeam technology effectively inactivated key BCO pathogens while preserving immunogenicity and safety. In this study, we combine the vaccine with two probiotics—GALLIPRO^®^ Hatch and GALLIPRO^®^ Fit, supplied by Novonesis (Hørsholm, Denmark). GALLIPRO^®^ Hatch, strategically formulated with *E. faecium*, is designed to establish gut health at hatch, while GALLIPRO^®^ Fit, containing *B. subtilis* and *B. amyloliquefaciens*, consistently promotes microbiome stability during growth. We hypothesized that the integration of GALLIPRO^®^ probiotics and the eBeam vaccine would have a synergistic effect in reducing BCO lameness. Using the Aerosol Transmission Model, a highly reliable method to induce BCO under controlled conditions [28], we evaluated the individual and combined effects of these interventions over a 56-day trial. By simultaneously leveraging both a vaccine and probiotics, this study aims to provide insights into sustainable strategies for combating BCO while improving bird health and productivity. The findings ultimately have the potential to benefit producers by offering practical, welfare-focused solutions to reduce lameness and optimize production efficiency.

## 2. Materials and Methods

### 2.1. Bacterial Culture and Vaccine Preparation

In this study, strains of *Staphylococcus agnetis*, *Staphylococcus aureus*, *Staphylococcus lentus*, and two types of *Staphylococcus cohnii* were isolated from lame chickens at our research farm during previous studies. These strains were stored at −80 °C and selected for vaccine preparation due to their predominant association with BCO lameness in broilers.

Each bacterial strain was individually cultured in 100 mL of Tryptic Soy Broth (TSB; Difco, Becton Dickinson, Sparks, MD, USA) and incubated at 37 °C overnight. The following day, the cultures were combined and centrifuged to remove the spent media. The resulting bacterial pellet was resuspended in fresh TSB to achieve a final concentration of approximately 1 × 10^8^ CFU/mL. This mixed bacterial suspension was then used for vaccine preparation as described below.

### 2.2. Electron Beam (eBeam) Vaccine Preparation

The electron beam irradiation process was conducted at the National Center for Electron Beam Research (NCEBR), located at Texas A&M University (TAMU) in College Station, TX, USA. A mixture of *Staphylococcus* strains (~1 × 10^8^ CFU/mL) was transferred into plastic bags, which were then heat-sealed to avoid leakage during shipping, in compliance with USDA-APHIS regulations. The bags, each containing 20 mL of bacterial culture, were sealed within a second set of plastic bags, which were also heat-sealed. These double-sealed bags were placed inside a leak-proof 95 kPa specimen transport envelope for safe handling. After sealing, the bags were sprayed with 70% ethanol and were dried with clean paper towels. The triple-bagged samples were then packed into a shipping box that met Department of Transportation and IATA guidelines and sent to NCEBR, TAMU. For the irradiation process, a high-energy linear accelerator (10 MeV, 15 kW) was employed. Alanine pellet dosimeters were positioned on both the top and bottom of each sample to measure the radiation dose absorbed. To ensure consistent irradiation, the dose-uniformity ratio was carefully controlled to be as close to 1.0 as possible. The samples received a dose of 8 kiloGrays (kGy) and were then stored at 4 °C after arrival. These irradiated samples were subsequently utilized as the eBeam vaccine.

### 2.3. Probiotic Preparation

#### 2.3.1. GalliPro^®^ Fit

GalliPro^®^ Fit (Novozymes, Hørsholm, Denmark), a commercial triple-strain Bacillus-based probiotic containing two *B. subtilis* strains (*B. subtilis* 597, *B. subtilis* 600) and one *B. amyloliquefaciens* (*B. amyloliquefaciens* 516), was used in this study. The product was supplied in powdered form with a guaranteed microbial concentration of 1.6 × 10^10^ CFU per g. The probiotic was diluted daily in drinking water to ensure uniform delivery to all broilers. The dosage administered ranged from 150 to 300 g per 5000 birds per day, corresponding to 5.0 × 10^8^ CFU per bird (for the 150 g dose) or 1.0 × 10^9^ CFU per bird (for the 300 g dose), in accordance with the manufacturer’s instructions. The probiotic dosage was maintained consistently throughout the experimental period without adjustment based on bird age or growth stage.

#### 2.3.2. GalliPro^®^ Hatch

The *E. faecium* strain (*E. faecium* 669) used in this study was sourced from the commercially available product GalliPro^®^ Hatch (Novozymes, Hørsholm, Denmark), containing a 2.0 × 10^11^ CFU/g concentration. Based on the manufacturer’s dosing recommendations, a concentration of 2.0 × 10^9^ CFU per chick was prepared and applied using an in-house static spraying system on day 0. Chicks were housed in groups of 60 and manually sprayed with multiple passes until the designated volume per dose (75 mL per 60 chicks) was fully applied. To ensure proper distribution and facilitate visualization of spray coverage, non-toxic blue food dye was added to the probiotic solution. As this probiotic was only administered at hatch (day 0), no dosage adjustments were required.

### 2.4. Egg Incubation and In Ovo Vaccination Procedure

A total of 2200 fertilized eggs, obtained from Aviagen North America parent stock (Huntsville, AL, USA), were placed into an incubator under guidelines approved by the Institutional Animal Care and Use Committee (IACUC). Incubation was carried out at a temperature of 99.6 °F and a relative humidity of 85%. Egg viability was assessed on the 10th and 18th days of incubation by candling, and eggs that were non-viable, damaged, or spoiled were removed.

On day 18, in ovo vaccination was performed on 900 of the viable eggs, while the rest were kept unvaccinated for other experimental treatments. The eBeam vaccine was prepared by diluting it in fresh TSB to a final concentration of approximately 1 × 10^7^ CFU/mL. To create the vaccine mixture, equal amounts of the vaccine were blended together. Prior to vaccination, the larger ends of the eggs were disinfected using Kimwipes soaked in 70% ethanol and carefully punctured with an 18-gauge needle. A tuberculin syringe with a 1 mL capacity, paired with a 25-gauge needle and a depth-limiting guard set at 3 cm, was used to inject the vaccine into the amniotic cavity. Once the injections were complete, the puncture sites were sealed using melted paraffin applied with cotton swabs. The vaccinated eggs were then transferred back into hatching chambers to continue incubation under controlled conditions of 98 °F and 85% relative humidity until they hatched.

### 2.5. Animals and Facility

The University of Arkansas Institutional Animal Care and Use Committee approved the study under protocol #24004. A total of 1560 one-day-old chicks were housed in 26 pens (1.5 m × 3.0 m each), with an initial stocking density of approximately 750 cm^2^ per chick. By day 14, the chick density in each pen was adjusted to 60 birds per pen, maintaining a target density of 900 cm^2^ per chick. The pens were randomly assigned to two rows of 13 pens each. The experimental facility was equipped with automated systems to control temperature, light cycles, and ventilation, using tunnel ventilation and evaporative cooling pads. The photo period was set to a 23L:1D light cycle, with light intensity maintained at 20 lux throughout the study. Temperature settings were 32.2 °C from days 1 to 3; 31 °C from days 4 to 6; 29 °C from days 7 to 10; 26 °C from days 11 to 14; and 23 °C from day 15 onward. Each pen had a dedicated water line connected to municipal water on one side and two feeders positioned on the opposite side. All water lines were disinfected by flushing with a diluted bleach solution prior to the start of the experiment.

### 2.6. Aerosol Transmission Challenge Model

The effect of treatment groups on BCO lameness was assessed using an aerosol transmission challenge model [28]. In this model, birds were allocated to two wire-floor pens designated as BCO source groups (“seeder birds”). These pens were positioned near the evaporative cooling pads at the front of the house, while four exhaust fans were placed at the opposite end to create consistent unidirectional airflow from the wire floor pens toward the litter floor pens. The dietary treatment groups were housed on wood-shaving litter floors, with buffer zones—empty spaces—between the seeder birds and treatment groups to prevent direct contact. The layout of the wire floors, litter floors, and exhaust fans is crucial for the effective operation of the aerosol transmission model.

### 2.7. Experimental Design

**Figure A1:**
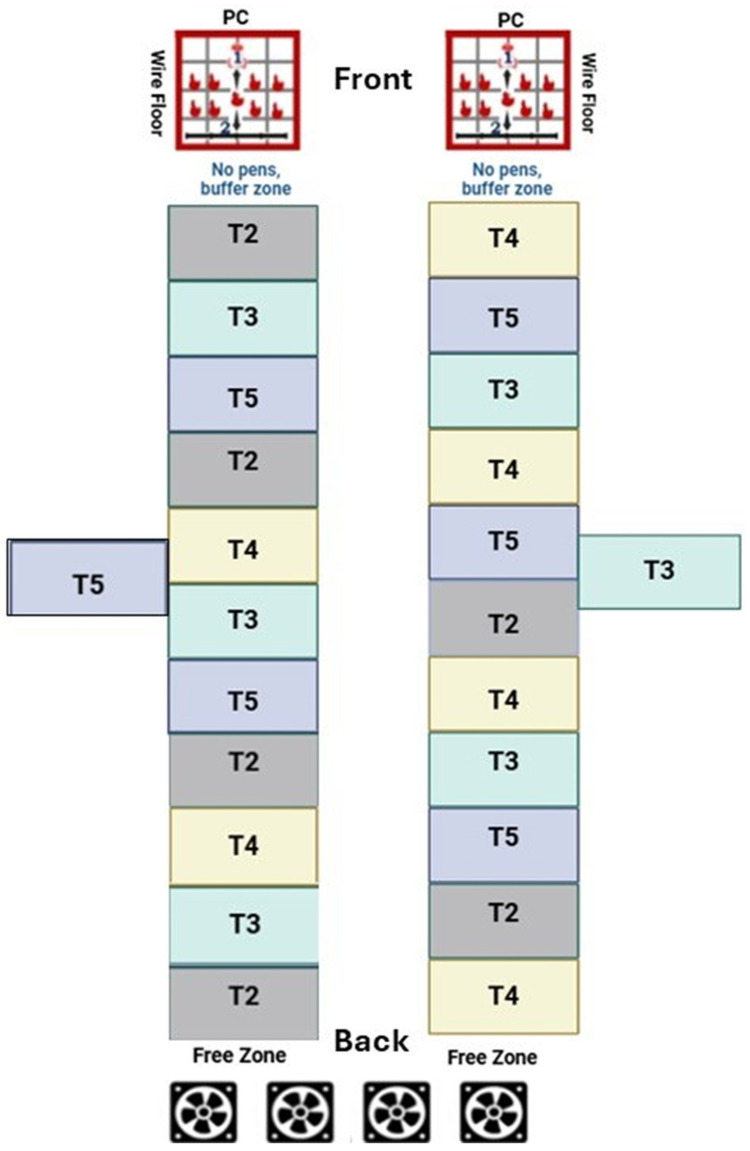
The experimental treatment layout diagram Treatments are marked as follows: PC = positive control, T2 = negative control, T3 = GALLIPRO^®^ Hatch/Fit, T4 = multivalent eBeam vaccine, and T5 = GALLIPRO^®^ Hatch/Fit and multivalent eBeam vaccine.

The experimental design employed a randomized block approach to ensure variability and representativeness across treatment groups. The study included five treatment groups, each with six pens, all housed on clean, dry wood-shaving litter floors, except for Treatment 1 (T1), where two pens were used as infection sources on wire floors. The wood shavings were regularly monitored and maintained to prevent excessive moisture buildup, ensuring optimal litter conditions for bird health and welfare. Treatment 2 served as a negative control, consisting of 6 pens, each housing 60 untreated birds. The remaining treatments (T3, T4, and T5) involved the inoculation of birds with various combinations of GALLIPRO^®^ Hatch and GALLIPRO^®^ Fit, and/or a multivalent eBeam vaccine. Birds treated with the multivalent eBeam vaccine were inoculated in ovo on day 18 of incubation according to standard industry protocols. GALLIPRO^®^ Hatch was sprayed on day-old chicks following Novonesis specifications, and GALLIPRO^®^ Fit was administered in drinking water from day 1 to day 56. A summary of the treatment groups is presented in Table 1, and the experimental treatment layout diagram is shown in Figure A1.

All birds were fed a standard diet, with the diet formulation presented in (Appendix A, Table A1), which was prepared at the University of Arkansas Poultry Research Feed Mill to meet commercial specifications based on Aviagen’s recommendations. All birds had access to water and feed ad libitum, provided as starter crumbles from D0 to D18, grower pellets from D18 to D42, and finisher pellets from D42 to D56.

### 2.8. Lameness Assessment

**Figure 1:**
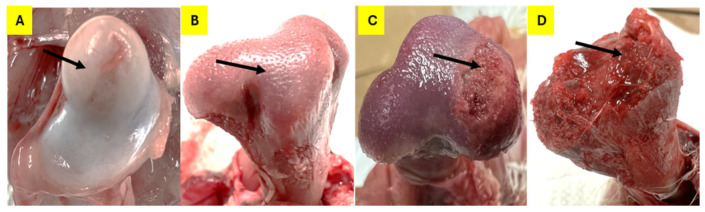
Classifications of femoral head lesions for BCO lesion development. The arrows indicate the affected regions in each stage of lesion progression: (**A**) N (normal); (**B**) FHS (femoral head separation); (**C**) FHT (femoral head transitional degeneration); (**D**) FHN (femoral head necrosis).

**Figure 2:**
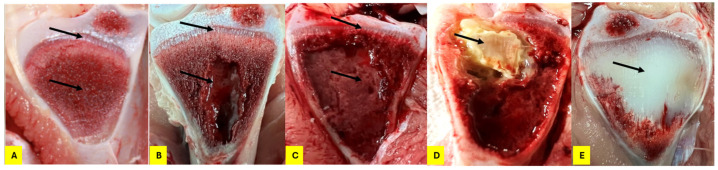
Classifications of tibial head lesions for BCO lesion developments. The arrows indicate the affected regions in each stage of lesion progression: (**A**) N (normal); (**B**) THN (tibial head necrosis); (**C**) THNS (tibial head necrosis severe); (**D**) THNC (tibial head necrosis caseous); (**E**) TD (tibial dyschondroplasia).

From day 22 to day 56, birds were monitored daily for signs of lameness by gently prompting them to move using standard kitchen brooms. Birds that exhibited reluctance to move or stand were marked with spray paint for further observation. Birds displaying difficulty walking, a goblet gait, or clinical signs of spondylitis/spondylolisthesis (e.g., sitting on their tails with extended legs, known as “kinky back”) were classified as clinically lame and euthanized for necropsy. All broilers that either died or developed clinical lameness were recorded by date and pen and underwent necropsy for gross examination. Tibial and femoral BCO lesions were evaluated and scored according to the classification system described by [4,9]. The total number of lame birds was calculated from those exhibiting at least one of the identified lesions. Figure 1 and Figure 2 depict the progression of these lesions.

### 2.9. Serotonin Analysis

On day 56, blood samples were collected from two birds per pen from treatments T2 to T5 for serotonin analysis. Blood was drawn into serum collection tubes following standard laboratory protocols. The samples were then processed and sent to a certified local laboratory, Whitebeck Labs (Springdale, AR, USA), for serotonin quantification according to their analytical procedures.

### 2.10. Body Weight Measurement

At the end of the trial (d56), six clinically healthy birds were randomly chosen to measure body weight and assess potential subclinical symptoms by categorizing tibial and femoral lesions.

### 2.11. Bacterial Sequencing

On day 56, five lame birds from each treatment group were selected for an aseptic necropsy to isolate bacterial cultures from tibial and femoral lesions. The protocol followed in this study was adapted from previously published methods [29,30]. The tissue samples were plated on three types of media: Difco™ Tryptic Soy Agar (Difco Laboratories, Franklin Lakes, NJ, USA), CHROMagar™ Orientation, and CHROMagar™ *Staphylococcus* (CHROMagar, Paris, France), and incubated at 37 °C for 24 to 48 h. Bacterial colonies were sorted by color and counted, with each colony re-streaked onto fresh plates. DNA extraction was performed using the DNeasy PowerLyzer Microbial Kit™ (QIAGEN, Hilden, Germany), following the manufacturer’s guidelines. The extracted DNA was stored at 4 °C until further use in PCR.

The 16S rDNA V1-V5 region was amplified using conventional PCR (cPCR) methods. The PCR mixture composition and cycling conditions are summarized in Table 2 and Table 3. PCR products were verified by 1.5% agarose gel electrophoresis using 0.5× TBE buffer at 60–70 V. The amplified DNA (40–60 ng/µL) and primers (2–10 pmol/µL) were sent to Eurofins Genomics Lab (Louisville, KY, USA) for sequencing. Sequence data were analyzed using the ApE Plasmid Editor software (https://jorgensen.biology.utah.edu/wayned/ape/, accessed on 2 June 2024) and queried against the National Center for Biotechnology Information (NCBI) database (https://www.ncbi.nlm.nih.gov/) using the Blastn tool. Species identification was confirmed when the sequence showed greater than 98% similarity to database sequences.

### 2.12. Statistical Analyses

The cumulative incidence percentage of lameness per pen and per treatment was analyzed using the *t*-test function in Microsoft Excel (Office 365, Microsoft Corp., Redmond, WA, USA), with the following formula:

$$\%\;\mathrm{cumulative}\;\mathrm{lameness}\;\mathrm{per}\;\mathrm{treatment}=\frac{\mathrm{Number}\;\mathrm{of}\;\mathrm{lame}\;\mathrm{bids}\;\mathrm{per}\;\mathrm{treatment}}{\mathrm{Total}\;\mathrm{number}\;\mathrm{of}\;\mathrm{birds}\;\mathrm{per}\;\mathrm{treatment}\;\mathrm{on}\;D14}\times 100$$

Treatment effects during the lameness trial (D14–D56) were assessed using logistic regression (binomial distribution) with the generalized linear model (GLM) procedure in R x64 2.4.2 (R Foundation for Statistical Computing, Vienna, Austria). Statistical significance was set at *p* < 0.05, and results are presented as mean ± SEM. The statistical unit for the analyses was the pen, as each treatment group consisted of multiple pens, and lameness was assessed at the pen level.

## 3. Results

**Figure 3:**
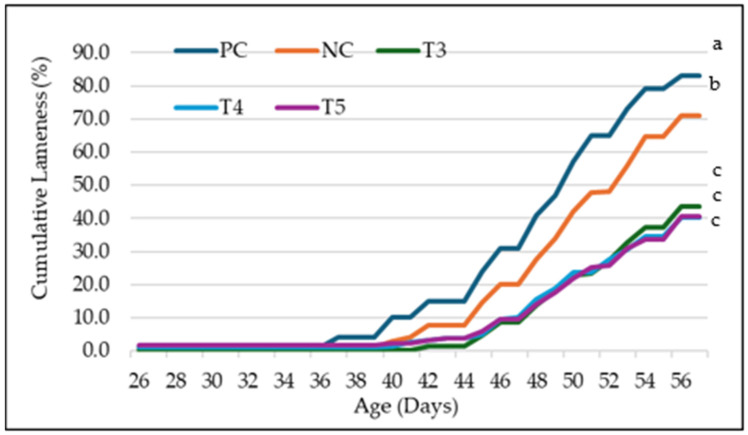
Percentage cumulative lameness by treatment groups from d36–57 of this study. (a–c) Values within a category with different letters vary significantly at *p* < 0.05 on day 56 (GLM analysis). Treatments are marked as follows: NC = negative control, PC = positive control, T3 = GALLIPRO^®^ Hatch/Fit, T4 = multivalent eBeam vaccine, and T5 = GALLIPRO^®^ Hatch/Fit and multivalent eBeam vaccine.

Cumulative lameness incidence varied significantly across treatment groups throughout the trial. Birds in wire flooring (PC) exhibited the earliest lameness onset on day 36 and the highest cumulative lameness incidence, peaking at 83% by day 56. The infection effectively spread to the negative control group of birds housed on litter flooring, reaching a final cumulative lameness incidence of 71%. In contrast, birds in the intervention groups—T3 (GALLIPRO^®^ Hatch/Fit), T4 (multivalent vaccine), and T5 (GALLIPRO^®^ Hatch/Fit-multivalent vaccine combination)—showed delayed lameness onset and significantly reduced cumulative lameness rates by day 56. Final lameness rates for T3, T4, and T5 were 43.7%, 40.3%, and 40.7%, respectively (*p* < 0.05), representing a marked improvement compared to the NC group. Notably, T5 maintained a consistently lower trajectory throughout the trial, although differences among treated groups narrowed by the end of the study (Figure 3).

Table 4 summarizes the cumulative lameness progression on the final weeks on days 35, 42, 49, and 56. The positive control (PC) group exhibited the highest lameness rates throughout the period, with cumulative incidences of 1.00%, 15.00%, 47.00%, and 83.00% on days 35, 42, 49, and 56, respectively. The negative control (NC) group showed the second-highest lameness rates, with incidences of 0.70%, 7.70%, 34.00%, and 71.00% on the same days. In contrast, the treated groups (T3, T4, and T5) exhibited significantly lower cumulative lameness rates than the control. On days 35, 42, 49, and 56, the incidences were as follows: T3 recorded 0.3%, 1.3%, 18.3%, and 43.7%; T4 recorded 1.00%, 3.30%, 19.00%, and 40.30%; and T5 recorded 1.70%, 3.30%, 17.70%, and 40.70%. Throughout this period, treated groups experienced a more gradual increase in lameness compared to the PC and NC groups. The weekly rise in lameness for treated groups ranged from approximately 5% to 20%, whereas the PC and NC groups showed sharper increases, particularly between days 42 and 56.

Table 5 presents the binomial logistic regression analysis results of cumulative lameness incidence among treatment groups on day 56. The PC group showed a significantly higher cumulative lameness incidence compared to T3 (*p* = 0.01), T4 (*p* = 0.00), and T5 (*p* = 0.01), but not when compared to the NC group (*p* = 0.05). The NC demonstrated significantly higher lameness rates than all treated groups, with *p*-values less than 1.0 × 10^−4^ when compared to T3, T4, and T5. Among the treated groups, no significant differences were observed between T3 and T4 (*p* = 0.09), T3 and T5 (*p* = 0.06), or T4 and T5 (*p* = 0.44), indicating similar efficacy in reducing cumulative lameness incidence.

**Figure 4:**
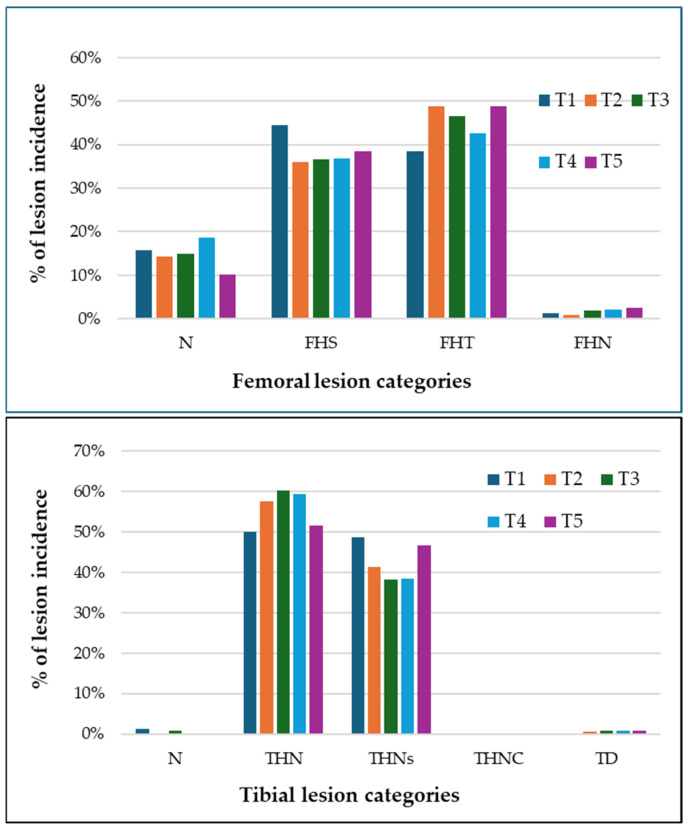
Distribution of femoral and tibial head lesion severity across treatment groups. Lesion categories, based on Wideman (2016) [2], include normal femoral and tibial head (N), femoral head separation (FHS), femoral head transitional lesions (FHTs), femoral head necrosis (FHN), tibial head necrosis (THN), tibial head necrosis severe (THNs), tibial head necrosis caseous (THNC), and tibial dyschondroplasia (TD). Treatment groups are labeled as follows: T1 = positive control, T2 = negative control, T3 = GALLIPRO^®^ Hatch/Fit, T4 = multivalent eBeam vaccine, and T5 = GALLIPRO^®^ Hatch/Fit combined with multivalent eBeam vaccine.

Figure 4 illustrates the distribution of femoral and tibial head lesion severity across treatment groups. Among the femoral lesion categories, femoral head separation (FHS) and femoral head transitional lesions (FHTs) were the most frequently observed. FHTs in the NC group exhibited the highest incidence rate at 50.23%. In contrast, femoral head necrosis (FHN) had the lowest occurrence across all treatments. For tibial lesions, tibial head necrosis (THN) and tibial head necrosis severe (THNS) were the most prominent categories. THN in T4 demonstrated the greatest incidence rate at 67.77%, emphasizing the severity of lesions in this group. Tibial dyschondroplasia (TD) and tibial head necrosis caseous (THNC) were rare, with minimal occurrences across all treatments. Treatments T3, T4, and T5 generally reduced the severity and frequency of tibial lesions. However, no clear trend was observed for femoral lesion severity across the treatment groups. Although certain treatments appeared to mitigate lesion severity, there was no consistent pattern of reduction in lesion severity between treatments.

**Figure 5:**
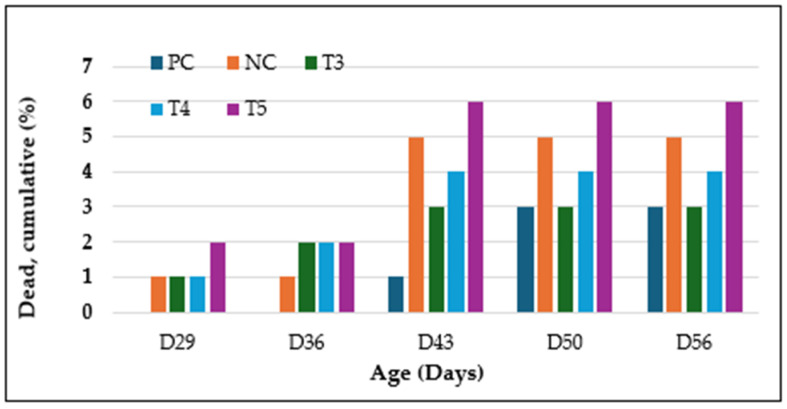
Weekly cumulative mortality across treatment groups. Treatment groups are labeled as follows: NC = negative control, PC = positive control, T3 = GALLIPRO^®^ Hatch/Fit, T4 = multivalent eBeam vaccine, and T5 = GALLIPRO^®^ Hatch/Fit combined with multivalent eBeam vaccine.

In addition, mortality was evaluated alongside the cumulative incidence of lameness during the final four weeks of the trial. Figure 5 shows the weekly cumulative mortality rates for each treatment group, focusing specifically on deaths classified as DUR (Dead Unknown Reason) and SDS (Sudden Death Syndrome). The T5 group had the highest cumulative mortality rate throughout the trial, followed by the NC and T4 groups. In contrast, the PC group had the lowest mortality rate due to lameness culls.

Table 6 summarizes the percentages of healthy birds, lameness, and mortality categories across treatment groups on day 56.

Table 7 highlights the average serotonin levels across the treatment groups at d56. The NC group exhibited the lowest serotonin level at 4155.20 ng/mL. In the treated groups, serotonin levels progressively increased, with T3 and T4 reaching levels of 4508.30 ng/mL and 4997.30 ng/mL, respectively. Notably, the T5 group, which combined GALLIPRO^®^ Hatch/Fit with the multivalent eBeam vaccine, demonstrated the highest serotonin levels at 7072 ng/mL.

On day 56 of age, the average body weights of six clinically healthy birds showed notable differences across treatments. Birds in the positive control group (PC) had an average weight of 4.28 ± 0.32 kg, while those in the T3 group (GALLIPRO^®^ Hatch/Fit) exhibited the highest average weight of 4.6 ± 0.15 kg. Birds in the T4 group (multivalent eBeam vaccine) weighed 4.42 ± 0.25 kg, and those in the T5 group (GALLIPRO^®^ Hatch/Fit combined with multivalent eBeam vaccine) showed the lowest average weight of 3.67 ± 0.71 kg (Table 8).

**Figure 6:**
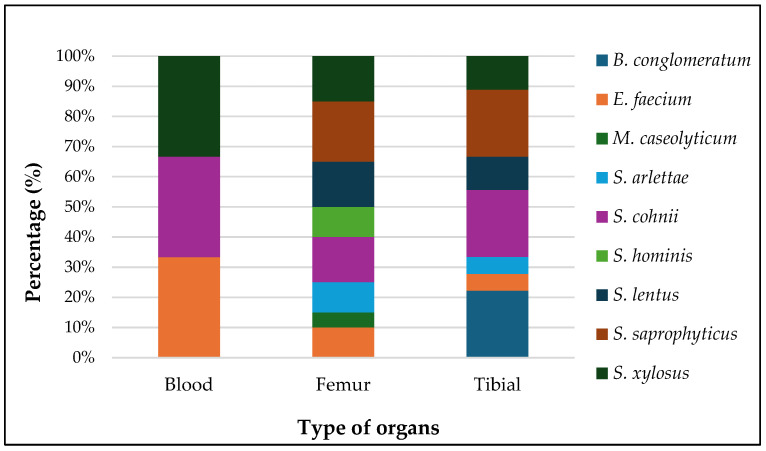
Distribution of bacterial identifications across different tissue types of lame birds in all treatments.

**Figure 7:**
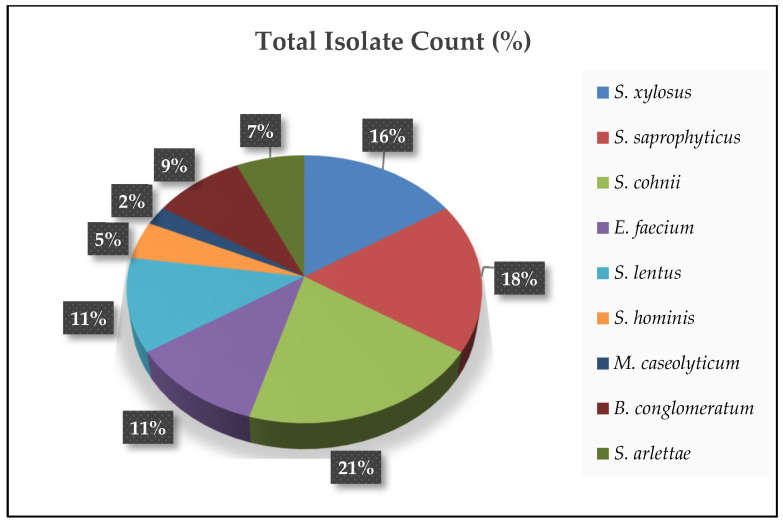
Total isolate count (%) of bacterial species isolated from blood, femur, and tibial of lame birds.

Nine types of bacteria were isolated from the blood, femur, and tibial of lame birds across all treatments, including *Staphylococcus* spp., *Enterococcus faecium*, *Brachybacterium conglomeratum*, and *Macrococcoides caseolyticum* (Figure 6 and Figure 7). The most abundant bacteria identified in this study were *Staphylococcus cohnii* (21.43%), *Staphylococcus saprophyticus* (19.05%), and *Staphylococcus xylosus* (16.67%). Other notable isolates included *E. faecium* and *Staphylococcus lentus*, each comprising 11.90% of the total isolates.

## 4. Discussion

This study investigated the effects of probiotic inclusion and the multivalent eBeam inactivated vaccine, both individually and in combination, on reducing BCO lameness in broiler chickens using an aerosol transmission model to induce BCO. The study demonstrated significant reductions in lameness incidence among intervention groups compared to the positive (PC) and negative control (NC) groups, emphasizing the efficacy of the tested strategies. In agreement with previous studies, the aerosol transmission model effectively triggered BCO in broilers housed in wire flooring pens, as evidenced by the early onset on day 36 of age and high cumulative lameness incidence in the PC group. Wire flooring has been extensively reported to exacerbate skeletal stress, induce microfractures, and compromise tight junction integrity, leading to increased bacterial translocation into the bloodstream and subsequent colonization of growth plates [4,28,29,31]. Similarly, airborne transmission of etiological agents to litter-flooring pens in the negative control group (NC) further validated the model’s capability to simulate field-like BCO outbreaks [29,31]. These findings underscore the robustness and reliability of the experimental model for studying BCO pathogenesis and intervention efficacy.

According to our findings, broilers in the treatment groups (T3, T4, and T5) consistently showed noticeably reduced rates of lameness than those in the negative control (NC) and positive control (PC) groups. It is evident that these interventions have significant potential to delay the occurrence of lameness and reduce its severity over time. Regardless of the specific treatment group, both probiotics and vaccines played distinct and complementary functions in mitigating bacterial chondronecrosis with osteomyelitis (BCO) lameness. More precisely, the eBeam vaccine group (T4) was instrumental in promoting pathogen-specific immunity, preventing bacterial colonization, and reducing systemic dissemination of opportunistic pathogens such as *Staphylococcus agnetis*, a predominant cause of BCO [27]. Systematically, electron beam (eBeam) irradiation effectively deactivates bacteria by damaging DNA while preserving the integrity of cell surface antigens, thus allowing the immune system to initiate a targeted response [27,32,33,34]. This method has been shown to demonstrate particular efficacy in ensuring enhanced antigenicity and provoking strong immunity, including increased mucosal responses via secretory IgA. These responses act to neutralize pathogens at mucosal surfaces and inhibit their transfer into the bloodstream, a key step in BCO pathogenesis [27,32,33,34]. The vaccine’s effectiveness in lowering lameness rates in our study corresponds with earlier research in which eBeam-killed *Staphylococcus* vaccines decreased lameness incidence by 50% through robust humoral and mucosal immune responses [27]. In contrast, probiotics played a crucial role in the protective effects noted in this research. These living microorganisms, such as *B. subtilis*, *B. amyloliquefaciens*, and *E. faecium*, enhance gut health by maintaining microbiota balance, strengthening intestinal barrier function, improving bone tissue integrity, and reducing systemic inflammation [35,36]. Probiotics strengthen gastrointestinal defenses by enhancing physical barriers like the mucus layer, epithelial cells connected by tight junction proteins (OCLN, CLDN-2, and ZO-1), and immune cells [18,31]. Despite environmental or pathogenic challenges, our findings revealed that the inclusion of these probiotics successfully diminished bacterial translocation, thereby alleviating BCO lameness. These findings align with earlier studies where probiotics such as Bacillus subtilis and Bacillus licheniformis significantly reduced lameness and improved intestinal integrity [37].

Furthermore, our earlier research indicated that administration of *E. faecium* to newly hatched chicks on day 0 effectively lowered cumulative BCO lameness by the end of the study [38]. Likewise, studies on lactic acid bacteria (LAB) probiotics have shown that they can boost the ratio of villus height to crypt depth, increase the expression of tight junction proteins, and enhance intestinal permeability, even when the body is under stress [39,40,41]. Taken together, these findings are consistent with our results and provide further support for the outcomes observed.

The analysis of the severity levels of femoral and tibial lesions presented in this research highlights the complex challenges faced in preserving bone integrity in broiler chickens. Among the femoral lesions, femoral head separation (FHS) and femoral head transitional lesions (FHTs) emerged as the most common types, whereas femoral head necrosis (FHN) was rarely observed across all treatment groups. Significantly, the NC (negative control) group showed the highest frequency of FHTs, reaching a peak occurrence of 50.23%. This observation aligns with previous studies that have indicated untreated populations tend to exhibit increased rates of FHTs, likely due to the lack of interventions designed to halt the progression of lesions [29,31,38]. A similar pattern was evident with tibial lesions, where tibial head necrosis (THN) and severe tibial head necrosis (THNS) were the most frequently noted categories. The highest rate of THN was recorded in the T4 group (multivalent eBeam vaccine), with an incidence of 67.77%, highlighting the severity of tibial injuries in this group. Despite observing reductions in lameness among the probiotic-treated groups (T3, T4, and T5), no consistent patterns in femoral lesion severity were detected across the treatment groups. This is consistent with earlier research suggesting that probiotic supplementation may lower the overall incidence of lameness without necessarily leading to significant improvements in lesion severity [31,38]. Likewise, while some treatments seemed to lessen the severity of tibial lesions, no clear trend of reduction was identified. This study’s progression of lesion severity reflects established pathophysiological models of bacterial chondronecrosis with osteomyelitis (BCO). Advanced lesions, such as FHN and THNS, indicate considerable pathological damage, including complete necrosis and extensive degradation of growth plate cartilage [42]. These lesions are linked to significant mobility challenges, hindering affected birds’ ability to access feed and water, thus compromising their overall health and welfare [43]. On the other hand, less severe lesion types like FHS and FHTs represent earlier phases of pathological development. Although they are less destructive, these lesions still lead to noticeable gait abnormalities and discomfort, underscoring the significant welfare concerns even at intermediate stages of lesion progression.

We observed a diverse composition of bacterial species isolated from the blood, femur, and tibia of lame birds across all treatment groups, underscoring the polymicrobial character of bacterial chondronecrosis with osteomyelitis (BCO) lesions. In this study, we identified *S. cohnii*, *S. saprophyticus*, *S. xylosus*, *E. faecium*, *B. conglomeratum*, and *M. caseolyticum*, further illustrating the complexity of BCO pathogenesis. The prevalence of *Staphylococcus* and *Enterococcus* species in this research corresponds with previous studies that recognized these genera as significant players in forming BCO lesions [29,30,31]. Key pathogens like *E. coli*, *Enterococcus* spp., *Serratia* spp., and Staphylococcus spp. are often associated with BCO, typically alongside secondary opportunistic bacteria such as *Salmonella* spp. [10,44,45]. Notably, *S. cohnii*—a species frequently isolated in this research—has recently been highlighted in genomic studies for its connection to osteomyelitis lesions in poultry, reinforcing its involvement in joint infections [46]. The repeated identification of *Staphylococcus* spp. across various studies, including nearly half of the isolates found here, reinforces their pivotal role in BCO progression [29,30,31]. These results highlight that BCO arises from the intricate interaction of numerous bacterial species, environmental factors, and host characteristics. Bacteria can be spread through multiple pathways, such as vertical transmission, aerosol inhalation, or contaminated feed or water consumption. Moreover, the specific distribution of bacterial species underscores their ability to target different tissues, thereby intensifying disease severity.

Finally, our findings also showed an increase in serotonin levels among the treatment groups, highlighting the capacity of dietary and vaccine strategies to influence this biomolecule associated with bone health and stress management. Remarkably, T5 exhibited the peak serotonin levels, implying a synergistic effect between probiotics and the eBeam vaccine compared to either treatment by itself. Conversely, the NC group displayed significantly lower serotonin levels, emphasizing the detrimental consequences in the absence of intervention measures. The reason for analyzing serotonin in this study is its essential role in gut health and bone metabolism. This observed increase in serotonin levels aligns with prior research illustrating the role of probiotics in enhancing gut health and nutrient absorption. *Bacillus subtilis* and related probiotics are known to promote the growth of beneficial gut bacteria while suppressing pathogenic strains [47]. This improved gut environment facilitates the absorption of tryptophan, the precursor to serotonin, which can subsequently increase serotonin synthesis [48,49]. Furthermore, probiotics modulate the gut microbiota to influence the production of short-chain fatty acids (SCFAs), which are positively correlated with serotonin production [50]. Consequently, the findings from the T3 and T5 groups are consistent with the established mechanisms of probiotics in serotonin modulation. Additionally, serotonin plays a critical role in bone health by influencing osteoblast and osteoclast activity, essential for bone formation and resorption [50]. Higher serotonin levels have been associated with improved bone mineralization, which is crucial for broiler growth and structural integrity [51,52]. The elevated serotonin levels observed in the T5 group suggest that the combination of probiotics and the eBeam vaccine enhances bone metabolism, likely through improved calcium and phosphorus utilization mediated by serotonin. Thus, serotonin plays a crucial role in connecting gut health, nutrient absorption, and bone strength, making it an important factor in improving poultry welfare and productivity.

Furthermore, dietary tryptophan supplementation has been linked to better skeletal health, reinforcing the role of serotonin as a mediator between dietary interventions and bone integrity [51,52]. Our results indicate that the combination of probiotics and vaccines promotes overall health, reduces stress, and enhances welfare outcomes in broilers. This dual strategy underscores the promise of integrated interventions for sustainable poultry production systems.

## 5. Conclusions

This study evaluated the efficacy of a probiotic program and a multivalent electron beam-inactivated vaccine in reducing BCO lameness in broilers using a wire-floor aerosol transmission model. All treated groups showed a significant reduction in cumulative lameness incidence compared to the untreated control, demonstrating the potential of these interventions. The *Enterococcus faecium* spray at hatch and the triple-strain *Bacillus* probiotic in drinking water effectively reduced lameness by approximately 30%, either alone or combined with the vaccine. These findings support using probiotics as a sustainable strategy to improve broiler welfare and reduce economic losses associated with BCO lameness. Further research is needed to explore the mechanisms underlying these effects, including their impact on gut health, immune responses, and bacterial colonization in bone tissues. Validation in commercial production systems will help establish these interventions as practical solutions for the broiler industry. This study contributes to advancing poultry health and supports the development of strategies to enhance animal welfare and sustainability.

## 6. Patents

A highly effective electron beam (eBeam)-killed multi-bacterial vaccine for mitigating broiler chicken lameness, elevating avian health, improving animal welfare and reducing financial loss”, Provisional Patent Application Filed.

## Figures and Tables

**Table 1:** Description of the treatment designations in the study.

Treatment	Flooring	Number of Pens Allocated
T1: Positive Control (PC)—Infection source	Wire	2
T2: Negative Control (NC)	Litter	6
T3: GALLIPRO^®^ Hatch/Fit	Litter	6
T4: Multivalent eBeam vaccine	Litter	6
T5: GALLIPRO^®^ Hatch/Fit and Multivalent eBeam vaccine	Litter	6

**Table 2:** PCR reaction components and their respective volumes.

PCR Component	Volume (µL)
Phusion^®^ High-Fidelity PCR Master Mix *	25.0
Forward primer (5′-AGAGTTTGATCCTGGCTCAG-3′)	0.5 µM
Reverse primer (5′-GTGCGGGCCCCCGTCAATTC-3′)	0.5 µM
Dimethyl sulfoxide (DMSO)	1.5
DNA template	2.0
Nuclease-free water (NFW)	20.5
Total Volume	50.0

* Thermo Fisher Scientific, Waltham, MA, USA.

**Table 3:** PCR cycling conditions for 16S rDNA amplification.

PCR Cycling Conditions	Temperature (°C)	Time	Cycles
Initial denaturation	98 °C	30 s	1
Denaturation	98 °C	10 s	35
Annealing	71 °C	30 s	35
Extension	72 °C	30 s	35
Final extension	72 °C	3 min	1
Hold	4 °C	∞	-

**Table 4:** Cumulative lameness progression per week from d35–56 (in %).

Day	PC	NC	T3	T4	T5	*p*-Value
35	1.00	0.70	0.30	1.00	1.70	>0.05
42	15.00	7.70	1.30	3.30	3.30	>0.05
49	47.00	34.00	18.30	19.00	17.70	>0.05
56	83.00	71.00	43.70	40.30	40.70	>0.05
±SEM	±25.80	±22.52	±14.35	±12.82	±12.77	

Treatments are marked as follows: NC = negative control, PC = positive control, T3 = GALLIPRO^®^ Hatch/Fit, T4 = multivalent eBeam vaccine, and T5 = GALLIPRO^®^ Hatch/Fit and multivalent eBeam vaccine.

**Table 5:** Binomial logistic regression of cumulative lameness incidence at d56.

*p*-Value	NC	T3	T4	T5
PC	0.05	0.01 *	0.00 *	0.01 *
T2		<1.0 × 10^−4^ *	<1.0 × 10^−4^ *	<1.0 × 10^−4^ *
T3			0.09	0.06
T4				0.44

Note: Asterisks (*) indicate statistical significance at *p* < 0.05. Treatments are marked as follows: NC = negative control, PC = positive control, T3 = GALLIPRO^®^ Hatch/Fit, T4 = multivalent eBeam vaccine, and T5 = GALLIPRO^®^ Hatch/Fit and multivalent eBeam vaccine.

**Table 6:** Percentage of healthy, dead, and lame incidence at d56 for each treatment.

Condition	NC	PC	T3	T4	T5
KB	0.00	0.30	0.00	0.00	0.00
DUR	1.00	1.30	0.70	0.30	1.00
SDS	2.00	0.30	0.30	1.00	1.00
LAME	82.00	70.30	43.70	40.00	40.00
SCULL	2.00	2.70	2.30	2.70	3.00
HEALTHY	13.00	25.00	53.00	56.00	55.00

Note: KB (kinky back) is one type of LAME, SCULL = culled for sample collection, DUR = Dead Unknown Reason, SDS = Sudden Death Syndrome. Treatment groups are labeled as follows: NC = negative control, PC = positive control, T3 = GALLIPRO^®^ Hatch/Fit, T4 = multivalent eBeam vaccine, and T5 = GALLIPRO^®^ Hatch/Fit combined with multivalent eBeam vaccine.

**Table 7:** The average serotonin levels across the treatment groups at d56.

Treatment	Group Description	Avg. Serotonin Level (ng/mL) ± SEM
NC	Negative Control	4155.20 * ± 366.01
T3	GALLIPRO^®^ Hatch/Fit	4508.30 * ± 353.60
T4	Multivalent eBeam vaccine	4997.30 * ± 500.07
T5	GALLIPRO^®^ Hatch/Fit and Multivalent eBeam vaccine	7072.00 * ± 359.65

Note: Asterisks (*) indicate statistical significance at *p* < 0.05.

**Table 8:** Average body weights of clinically healthy birds in all treatments (in kg) on d56 of age.

Treatment	Number of Birds	Average Weight (±SEM, in kg)
PC	6	4.28 ± 0.32
T3	6	4.6 ± 0.15
T4	6	4.42 ± 0.25
T5	6	3.67 ± 0.7

Note: *p* > 0.05; there is no statistically significant difference in the mean body weights among the four treatments. PC = positive control, T3 = GALLIPRO^®^ Hatch/Fit, T4 = multivalent eBeam vaccine, and T5 = GALLIPRO^®^ Hatch/Fit combined with multivalent eBeam vaccine.

## Data Availability

The dataset used and/or analyzed in the study is available from the corresponding author upon request (A.A.K.A.).

## Appendix A

**Table A1:** Composition of the basal diets.

Ingredient Name	Starter %	Grower %	Finisher %
Corn, ground	47.46	55.82	62.31
Soybean meal	32.72	28.42	25.31
DDGS 28.4% UARK	13.18	8.96	5.83
Oil, poultry fat	3.34	3.66	3.76
Dicalcium phosphate	1.01	0.94	0.83
Limestone, fine	0.90	0.89	0.78
Dl-methionine 99%	0.30	0.29	0.30
L-LYSINE hcl, 78.8%	0.28	0.26	0.25
Na bicarbonate	0.28	0.25	0.21
Salt	0.16	0.17	0.18
L-threonine, 98%	0.11	0.10	0.09
Tyson 2x vitamin premix	0.10	0.10	0.05
U of A, poultry TM premix	0.10	0.10	0.05
Coccidiostat	0.05	0.05	0.05
Proximate analysis of broiler diets in the study			
Ash, %	4.98	4.66	4.08
Crude fat, %	4.03	4.36	5.39
Dry mater, %	89.9	90.5	88.8
Protein, %	25.7	23.1	19.9
Al, ppm	33.1	28.7	27.2
Ca, ppm	6645	6225	5384
Cu, ppm	25.2	19.4	20.1
Fe, ppm	168	129	101
K, ppm	9629	8319	7445
Mg, ppm	1875	1628	1472
Mn, ppm	112	105	62
Na, ppm	1034	1048	1061
P, ppm	6990	6136	5444
S, ppm	3365	2957	2185
Zn, ppm	123	121	82.3
